# Activation of the orbitofrontal cortex by both meditation and exercise: A near-infrared spectroscopy study

**DOI:** 10.1371/journal.pone.0247685

**Published:** 2021-02-23

**Authors:** Shun Miyashiro, Yurika Yamada, Toshizumi Muta, Haruyuki Ishikawa, Tetsuri Abe, Masashi Hori, Kotaro Oka, Fusako Koshikawa, Etsuro Ito

**Affiliations:** 1 Department of Biology, Waseda University, Tokyo, Japan; 2 Department of Psychology, Waseda University, Tokyo, Japan; 3 Department of Educational Psychology, Waseda University, Tokyo, Japan; 4 Department of Bioscience and Informatics, Keio University, Yokohama, Japan; 5 Waseda Research Institute for Science and Engineering, Waseda University, Tokyo, Japan; 6 Graduate Institute of Medicine, Kaohsiung Medical University, Kaohsiung, Taiwan; Tokai University, JAPAN

## Abstract

In some types of meditation, such as mindfulness and Zen, breathing is the focus of attention, whereas during an excessive, short-period of anaerobic exercise, the muscles become the focus of attention. Thus, during both efforts, one’s attention is focused on a certain feature of the body. Both meditation and exercise generally provide mental refreshment to humans. We hypothesized that the same brain regions are activated by both efforts in humans. To examine this hypothesis, we engaged participants in 3 tasks: meditation, exercise, and a control task. After each task, the participants underwent a 2-back test to concentrate their thoughts, while changes in their blood hemoglobin levels were simultaneously monitored using near-infrared spectroscopy (NIRS). Seventeen participants (20–24 years of age; 11 men, 6 women) were enrolled. We applied a fast-Fourier transform (FFT) analysis to the NIRS wave data and calculated the correlation coefficients of the FFT data between (1) meditation and control, (2) exercise and control, and (3) meditation and exercise, at the orbitofrontal cortex (OFC) and dorsolateral prefrontal cortex (DLPFC), brain areas that are generally involved in mental refreshment. A significant difference in the correlation coefficients between the OFC and DLPFC was detected in the meditation and exercise analysis, and signal source analysis confirmed that the NIRS waves spread from the right and left OFC edges (i.e., right and left temples) toward the center. Our results suggest that both meditation and exercise activate the OFC, which is involved in emotional reactions and motivation behavior, resulting in mental refreshment.

## Introduction

Humans obtain mental refreshment from meditation and exercise. Mental refreshment is provided by switching one’s attention to a certain feature (*e*.*g*., breathing, thought, body part *etc*.) [1–4]. In fact, mental disorders can be relieved by meditation and exercise [5, 6]. Meditation helps to ameliorate anxiety and depression, resulting in neurobiologic changes in the brain [7]. Attention-deficit/hyperactivity disorder (ADHD), which is associated with cortical thinning, is improved by meditation, which increases cortical thickness [8]. Posttraumatic stress disorder (PTSD) symptoms are also effectively attenuated by meditation [9]. Exercise, too, contributes to the management of mental disorders [10]. Exercise interventions can positively affect PTSD symptoms through modulating internal arousal cues and reducing inflammatory markers [11].

Certain types of meditation, such as mindfulness meditation, actively focus the practitioner’s attention on breathing [12, 13]. Mindfulness is the practice of being aware of one’s body’s sensation, thoughts, and feelings in the present moment. For example, in the neuroscience field, mindfulness meditation practices have come to the attention of many researchers investigating consciousness and affect regulation through mental training and to psychotherapists interested in personal development and interpersonal relationships [14]. On the other hand, an intensive, short-period anaerobic push-up exercise passively focuses the practitioner’s attention on their muscles. Both efforts lead to concentration on one feature of the body. We therefore hypothesized that when attention is focused during meditation or exercise, whether actively or passively, the same brain regions are activated because mental refreshment can be obtained by both efforts. Near-infrared spectroscopy (NIRS) is considered a suitable method for identifying active brain regions [15]. NIRS is a noninvasive neuroimaging method that measures oxy- and deoxy-hemoglobin concentration changes at the brain surface in real-time [16].

To test our hypothesis, we noted the 2 brain regions, the orbitofrontal cortex (OFC) and the dorsolateral prefrontal cortex (DLPFC), and examined whether the effects of meditation and exercise on these regions are similar. The OFC is a region of the prefrontal cortex that contributes to decision-making [17]. The DLPFC is located above the OFC and is most typically associated with executive functions, including selective attention [18]. Chronic and acute stress suppress the function of the prefrontal cortex, including the OFC and DLPFC [19]. Acute and uncontrollable stress evokes an increase in catecholamine release in the prefrontal cortex [20–22], thereby decreasing neuronal firing and cognitive function [19]. When participants look at images that induce mental stress, the activity in the DLPFC is suppressed; such stress is reduced by meditation [23]. Exercise, too, has long been thought to relieve stress [24]. There are some reports showing the effects of meditation on exercise outcomes [25, 26]. Furthermore, some recent studies suggested that the prefrontal cortex is activated by meditation and exercise [27, 28].

## Materials and methods

### Ethics statement

This study was carried out in accordance with the recommendations of the principles and guidelines of the Declaration of Helsinki. All participants provided their written informed consent to participate in this study. The protocol was approved by Waseda University, Office of Research Ethics (2016–282, 2018–067, 2019–358). The individual in this manuscript has given written informed consent (as outlined in PLOS consent form) to publish these case details.

### Participants

Seventeen voluntary participants who declared themselves to be mentally and physically sound on the basis of self-assessment (age 20–24 years; 11 men, 6 women; 2 left-handed and 15 right-handed) from the Tokyo area of Japan were enrolled. To ensure the participants’ sound mental condition, (1) we confirmed that none of the participants self-reported a history of neurologic or psychiatric disorders, or had a disease requiring medical care; and (2) we administered Center for Epidemiologic Studies-Depression Scale (CES-D) tests to exclude persons with an extraordinary pattern of cerebral blood flow [29]. The CES-D is useful for diagnosing depression [30]. This protocol has been widely used in previous studies [31–33]. We set the cut-off point as the scores of CES-D higher than 16 [34]. The mean and SD value of the CES-D for the participants was 9.80 ± 4.17. In the present study, all 17 participants were determined to be in a mentally and physically sound condition.

### Tasks

**Fig 1:**
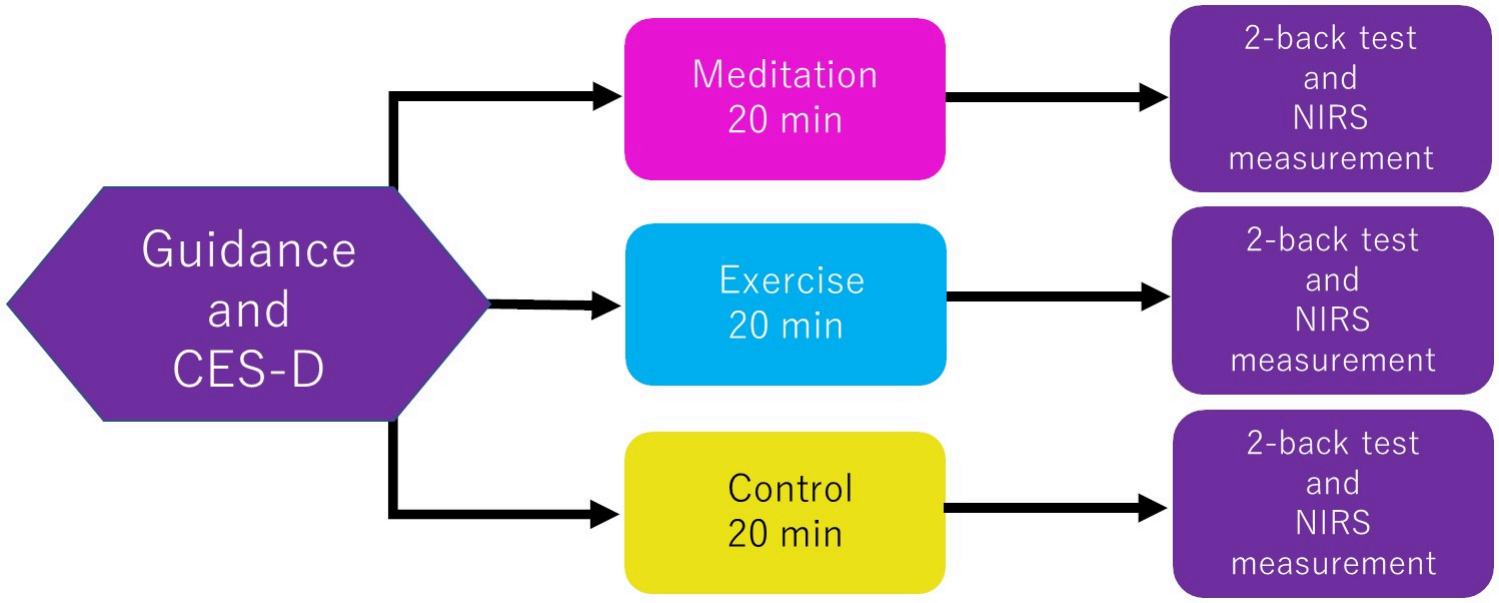
Experimental protocol. First, the CES-D was administered to the participants, and then 3 tasks: meditation, exercise, and a control task, were performed by the participants in a randomly selected order. After each task, a 2-back test was administered while the NIRS measurement was simultaneously performed.

During the tasks, the room temperature was controlled at 22–23°C. The room was kept very quiet and surrounded by the white walls. The following 3 tasks were performed by the participants: meditation, exercise, and a control task, 1 of each was carried out on a different day for a total of 3 days. The order of the tasks was randomly selected for each participant and counter-balanced across participants. In the meditation task, the participants were instructed to sit on a chair, keep their eyes open, and breathe 3 to 4 times a minute for a total of 20 min. They were also asked to pay attention to their respiration. In the exercise task, the participants performed push-ups for no more than 5 min. If the participants felt that it was impossible to continue the push-ups for the entire 5 min, the set was terminated. That is, based on the Rating of Perceived Exertion (RPE) scale [35], we instructed the participants to do push-ups until they felt very tired. After that, they could rest for the remaining time. They repeated this cycle 4 times and the total period was 20 min. The room was large enough to perform the push-ups. Approximately 8 to 23 push-ups were achieved during this period, and thus we considered this an anaerobic exercise. The inter-set interval was 5 min, and 4 sets of pushups were performed. In the control task, participants sat on a chair and watched a movie of scenery with relaxing music for 20 min. After each task, the participants underwent a 2-back test comprising 32 questions while simultaneously undergoing NIRS measurements (Fig 1).

In the 2-back test, the numbers (1–9) were displayed one after another randomly on the monitor in front of the participant. The participant answered the number displayed 2 steps earlier in the sequence on the monitor using the keyboard. When the participant answered, the next number was displayed. The 2-back test was performed to let the participants concentrate on something in order to stabilize their eyes and their body movements and then to stabilize their thoughts. Because the order in which each participant underwent the meditation, exercise, and control experiments was randomly selected, any potential learning effect on the 2-back test was canceled out across the cohort in the present study.

### NIRS measurement

**Fig 2:**
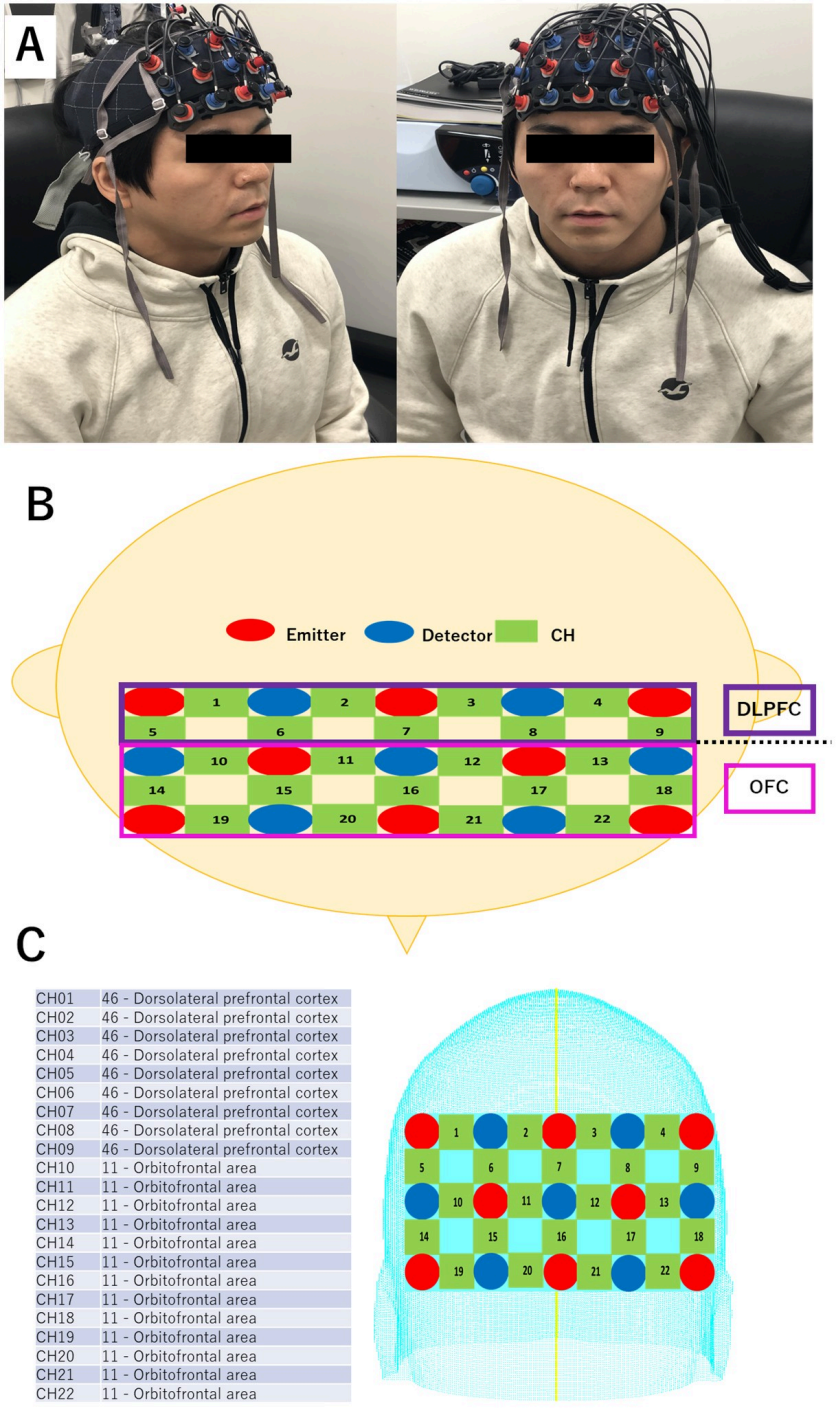
NIRS measurement. ***A***. A typical picture of a participant equipped with NIRS optodes. ***B***. The NIRS comprised 8 elements for the NIR light emitter (red) and 7 elements for the detector (blue). The numbers indicate the channels (green) recorded. The NIRS channels were placed above the dorsolateral prefrontal cortex (DLPFC) and the orbitofrontal cortex (OFC). ***C***. The channels between the optodes measured on the skull were assigned to the brain regions using the virtual registration method.

NIRS (ETG-4000/OT-R40, Hitachi, Tokyo, Japan) was used to detect blood flow over the DLPFC and the OFC. NIRS channels (CH) 1–9 were assigned to the DLPFC, and CH 10–22 were assigned to the OFC (Fig 2A and 2B).

The NIRS data is the measured relative concentration of oxyhemoglobin. The sampling rate of the NIRS was 10 Hz. All NIRS data were processed using high band-pass (0.001 Hz) and low band-pass (1 Hz) filters to remove artifacts, such as heartbeat and body movements. The NIRS optodes were placed in the proper position for each participant according to the international 10–20 system [36]. This system shows the location of scalp electrodes (i.e., NIRS optodes), particularly in electroencephalography (EEG) researches. This method ensures that human subject’s studies can be compiled, reproduced, analyzed and compared well each other. The numbers, ‘10’ and ‘20’, refer to the fact that the actual distances between adjacent optodes are either 10% or 20% of the total front-back or right-left distance of the skull. The points (*i*.*e*., channels) between the optodes measured on the skull were assigned to the brain regions using the virtual registration method [37, 38] (Fig 2C). We note that the OFC consists of the Brodmann areas 10 and 11 [39]. The responses in OFC can be measured with NIRS, because the OFC, at least the part of this region, is located within 3 cm under the skull [40–42]. The NIRS data were analyzed with MATLAB (2018b, MathWorks, Natick, MA, USA). Fast-Fourier transform (FFT) was applied to the NIRS data to analyze the frequency distribution of the NIRS data. To minimize the edge effects, we used the rectangular window for FFT, which was prepared as a default in MATLAB. Correlation coefficients of the FFT-analyzed NIRS signal frequencies were calculated among each pair of tasks, meditation-exercise, meditation-control, and exercise-control, for each channel of the DLPFC and OFC. After finding some channels on the left and right temples in which there were significantly larger correlation coefficients for the meditation and exercise pair than the other pairs, we performed signal source analysis as described below.

### Signal source analysis

We observed CH14 and CH18 at the right and left OFC edges (right and left temples, respectively). Using continuous wavelet transformation, we obtained the maximum correlation coefficients at the time delay of τ between each CH and CH 14/CH18. The time delay was obtained by continuous wavelet transformation. If the delay time was less than 0.5 s, and it increased associated with the distance from CH14 and CH18, we decided that the signals spread from CH14 and CH18 to the other regions. We mapped the time for each channel at which the peak values of the correlation coefficients in the meditation-exercise pair emerged within 0.0 to 0.5 s after the peak emergence at CH14 or CH18. We then calculated the cross-correlation values between CH14 or CH18 and another channel every 0.1 s, which was the NIRS resolution time. When the maximum value of this cross-correlation was obtained in a channel during the period of 0.1 to 1 s, the delay time was plotted. If the delay time increased as the distance increased from CH14 or CH18, then that channel (CH14 or CH18) was judged to be the signal source.

### Statistical analysis

The relation between the correlation coefficients of DLPFC and those of OFC is expressed in a box plot. This relation was analyzed by a Mann-Whitney *U*-test after an *F* test for equal variance. The relation between the correlation coefficients in each of the channels was analyzed as follows. The mean value of the correlation coefficients was subtracted from the correlation coefficients obtained for the meditation, exercise, and control tasks, and the value was referred to as a ‘correlation value’. In each channel, one-way analysis of variance (ANOVA) followed by a *post-hoc* Tukey test was performed to assess any differences in these correlation values. The Mann-Whitney *U*-test and ANOVA were performed with JSTAT for Windows (ver. 22.0J). A *p* of less than 0.05 was considered to indicate a significant difference. The 95% confidence interval (CI) was calculated using the Clopper-Pearson exact confidence interval method. The difference between groups was considered significant when the 95% CI of one group did not overlap with that of another group [43].

## Results

### NIRS signals and FFT-analyzed frequency

**Fig 3:**
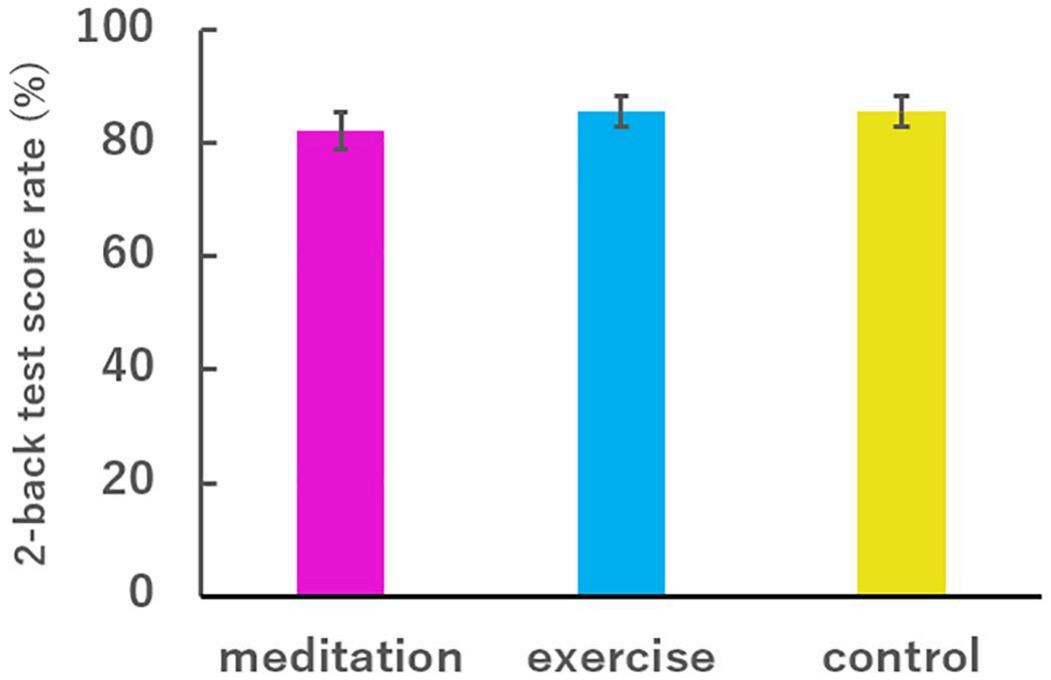
Scores (correct answer rate) in the 2-back tests. All participants performed the 2-back tests after meditation (red), exercise (blue), and control (yellow) tasks. The scores are shown as mean ± SEM. There is no significant difference.

We first found that the performance scores of the participants in the 2-back test were not significantly different following the meditation, exercise, and control tasks (Fig 3).

**Fig 4:**
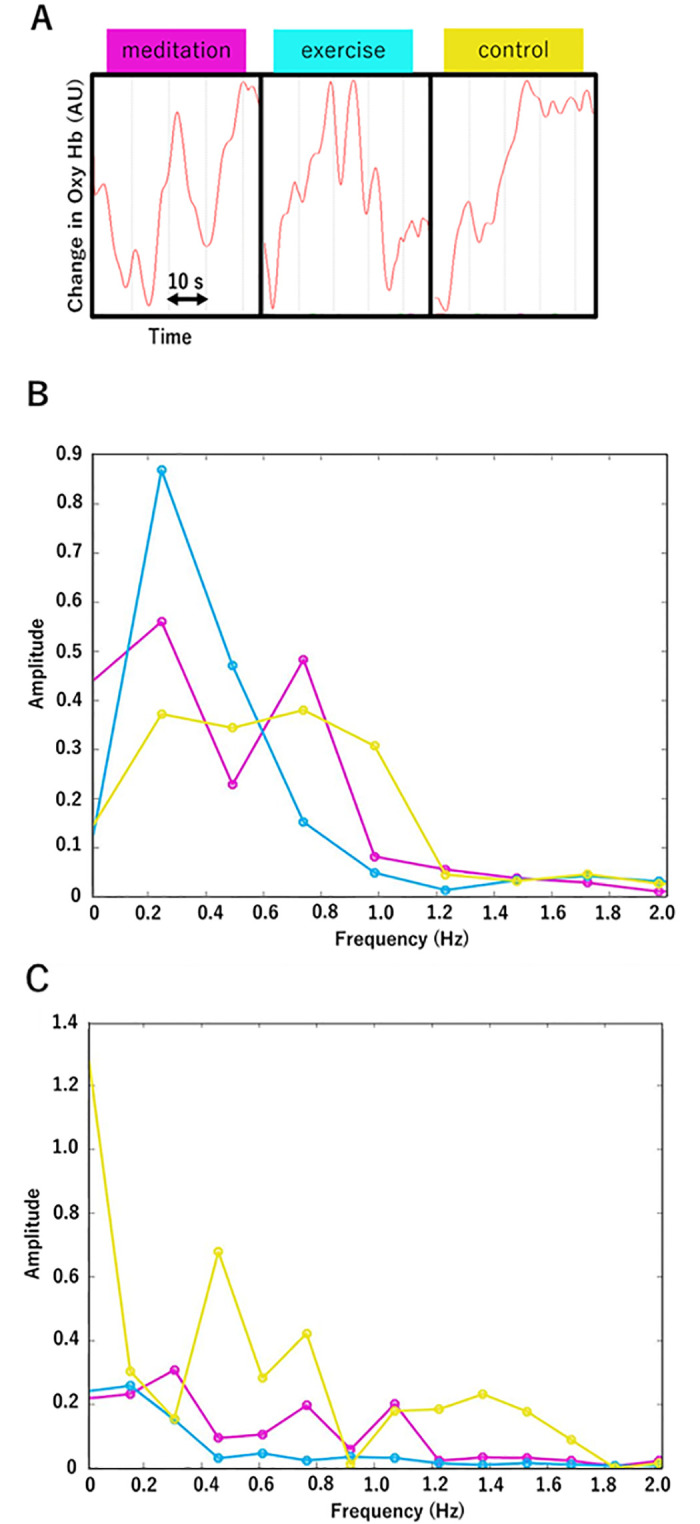
Representative NIRS signals and their FFT data. ***A***. At CH14 in a participant, the oxyhemoglobin NIRS signals (red lines) were obtained during the 2-back tests after meditation, exercise, and a control task. These figures show only typical signal changes as examples. ***B***. FFT values calculated from the data of a left-handed participant. ***C***. FFT values calculated from the data of a right-handed participant. Red line indicates the meditation data; blue line indicates those of exercise; and yellow line indicates those of control.

The oxyhemoglobin concentration changes measured with NIRS are thought to correspond to neuronal activity [44, 45]. Thus, relative concentrations of oxyhemoglobin were measured by NIRS during the 2-back tests after the meditation, exercise, and control tasks (Fig 4A).

**Fig 5:**
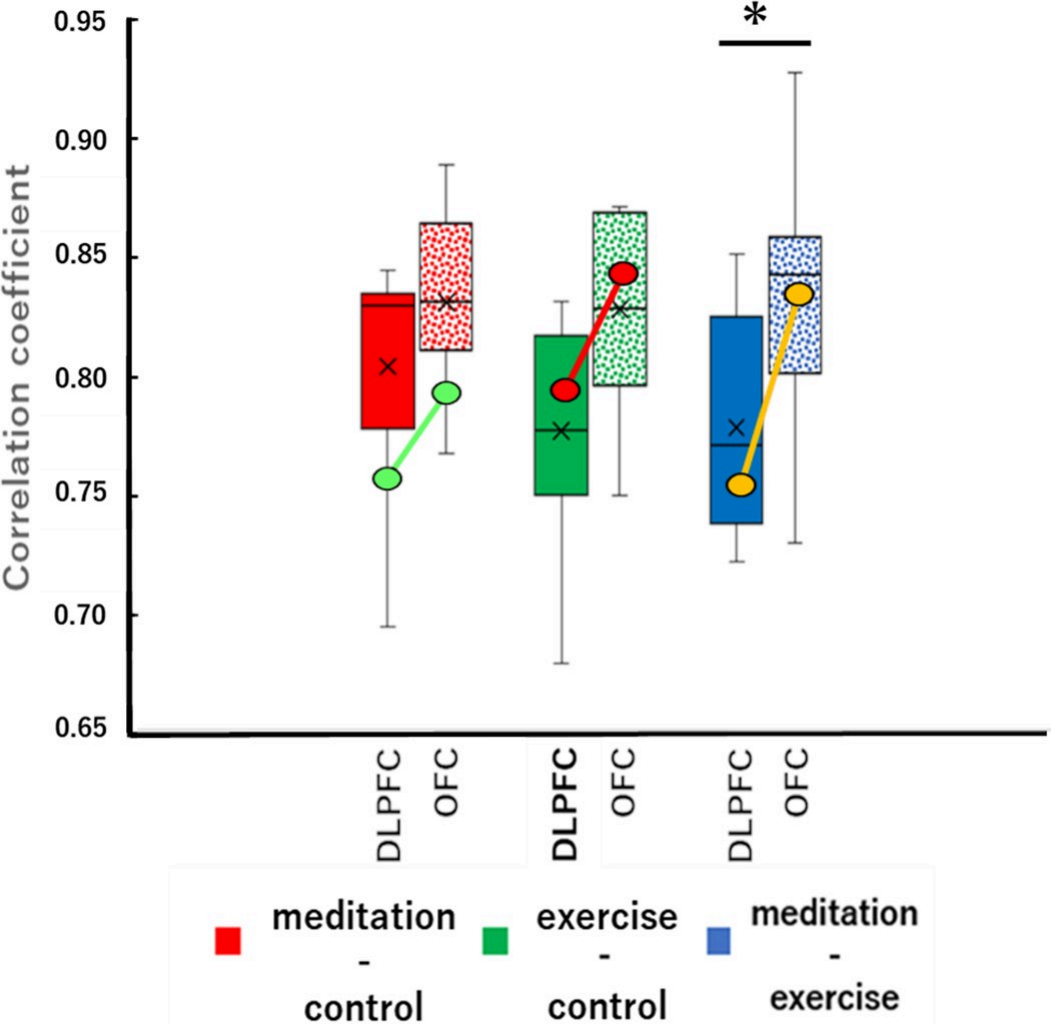
Correlation coefficients of the FFT-analyzed NIRS frequency at the DLPFC and the OFC for the meditation-control pair (red), the exercise-control pair (green), and the meditation-exercise pair (blue). A Mann-Whitney *U*-test was performed after an *F* test for equal variance. *p < 0.05. The diagonal lines show the changes in mean values obtained from the 2 left-handed participants.

The NIRS wave data exhibited large individual differences. We therefore analyzed the NIRS data by FFT and compared the spectra of the FFT data to overcome individual differences. Further, we compared the results of the 2 left-handed participants with those of the right-handed participants (n = 15) (Figs 4B, 4C and 5).

As these figures indicate, the results showed almost the same trend. Thus, we combined the results obtained from the left-handed participants and right-handed participants.

### Correlation coefficients of FFT-analyzed NIRS frequency in the DLPFC and OFC

Although the raw NIRS data appeared too varied to compare between tasks, the FFT data could easily be compared. Thus, the correlation coefficients of the FFT data were calculated and collected for the DLPFC and OFC (Fig 5). The results demonstrated that the correlation coefficients in the meditation-exercise pair were significantly larger in the OFC than in the DLPFC (*p* < 0.05).

**Fig 6:**
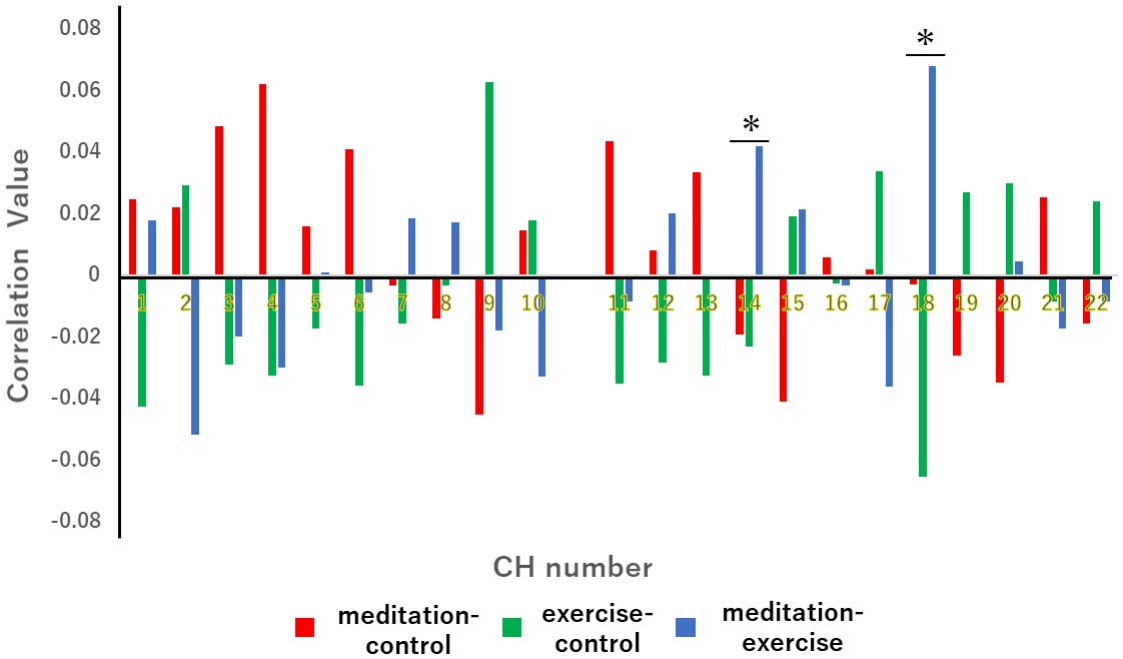
Coefficient correlations of FFT-analyzed NIRS frequencies at each channel among the meditation-control pair (red), the exercise-control pair (green), and the meditation-exercise pair (blue) analyses. The CH14 and CH18 showed significant differences only in the meditation-exercise pair (blue). One-way analysis of variance (ANOVA) followed by a *post-hoc* Tukey test was performed. For CH14, *F*(2,48) = 3.407, *p < 0.05; for CH18, *F*(2,48) = 3.207, *p < 0.05.

As the next step, the correlation coefficients of the FFT-analyzed NIRS frequencies at each channel were compared among the 3 pairs of tasks: meditation-control, exercise-control, and meditation-exercise. For this purpose, we calculated the ‘correlation values’ (see Materials and methods). At CH14 and CH18, which were the right and left OFC edges (*i*.*e*., right and left temples), the correlation coefficients were significantly larger for the meditation-exercise pair than for the meditation-control and exercise-control pairs [CH14: *F*(2,48) = 3.407, *p* < 0.05; CH18: *F*(2,48) = 3.207, *p* < 0.05] (Fig 6).

Thus, the edges of the OFC seem to play an important role in the brain activity that occurs during both meditation and exercise.

### Signal source analysis

**Fig 7:**
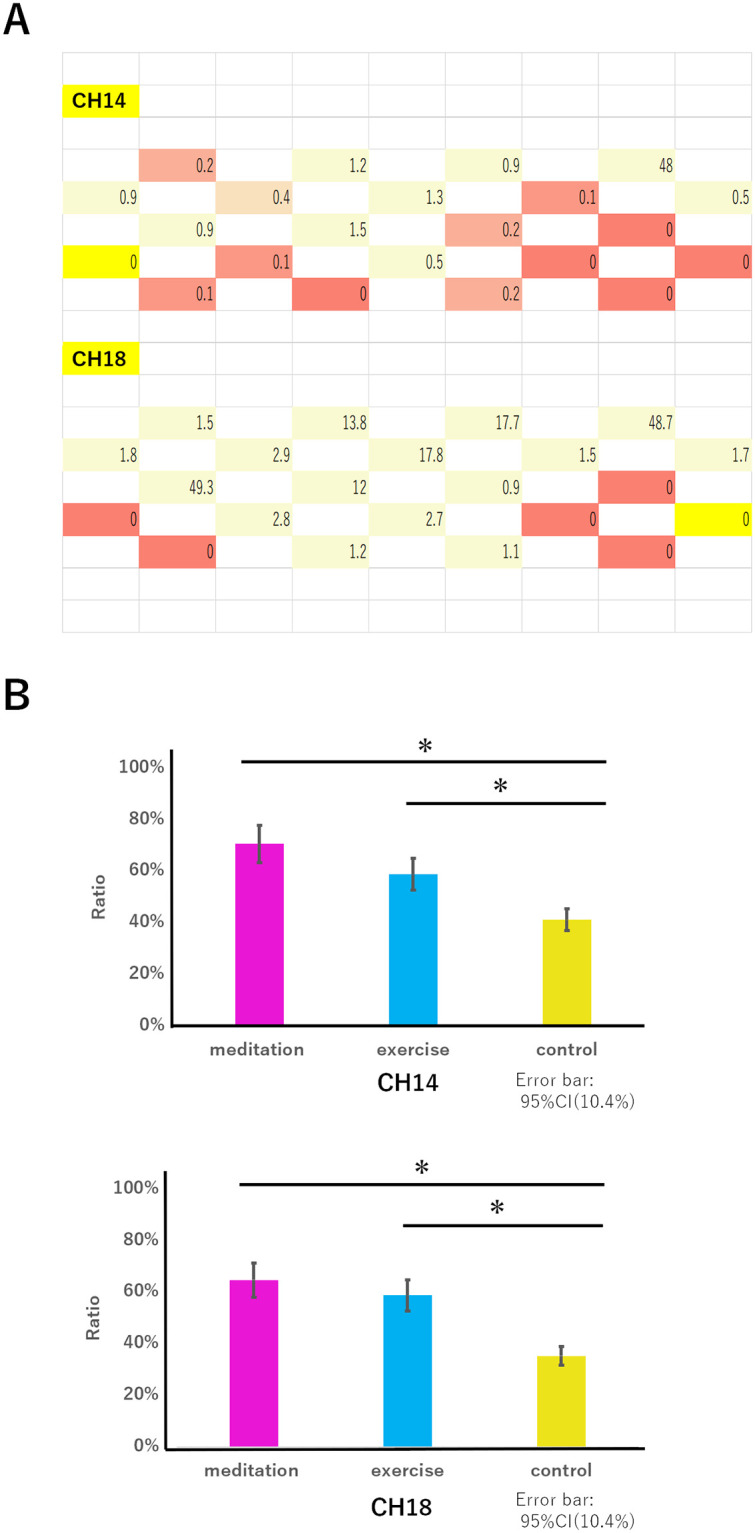
Signal source analysis for CH14 and CH18. ***A***. Representative data of spread of signals originating in CH14 and CH18 are shown. Numbers indicate the time at the maximum peak value of the correlation coefficients after the peak emergence of CH14 or CH18. ***B***. Ratios of CH14 or CH18 recognized as the signal source are shown for the 17 participants. Error bars indicate 95% CI (*e*.*g*., 10.4% for both CH14 and CH18), and if the error bars did not overlap, we determined that there was a significant difference between the tasks. * indicates a significant difference.

We mapped the time at each of the channels where the peak values of the correlation coefficients in the meditation-exercise pair emerged within 0.0 to 0.5 s after the peak activity emerged at CH14 or CH18 (Fig 7A).

The results obtained from the typical data (*i*.*e*., 1 participant) showed that the oxyhemoglobin concentration changes (*i*.*e*., a wave of neuronal activity) spread from the right and left OFC edges (*i*.*e*., right and left temples) to the midline of the brain. As far as we measured, this spread was observed once, not repeatedly, during each measurement. We then calculated the cross-correlation values between CH14 or CH18 and another channel every 0.1 s. When the maximum value of this cross-correlation was obtained in a channel, the delay time was plotted. A delay of more than 1 s was not observed in the NIRS imaging and wave data. Among the 17 participants, nearly 70% of those performing the meditation task and nearly 60% of those performing the exercise task showed this trend, whereas less than 40% of those performing the control task showed this trend (Fig 7B). The 95% CI was 10.4% for CH14 and 10.4% for CH18.

## Discussion

Our findings demonstrated that both meditation and exercise activate the right and left OFC edges (*i*.*e*., right and left temples), and the signal waves spread from these 2 specific sites toward the midline in the OFC. Our results seem reliable because (1) the OFC is involved in emotional reaction and motivation behavior [46]; and (2) the insular cortices, which are involved in both meditation and exercise [47–49], locate under the temples [50]. In particular, the change in insular cortices has been vigorously examined in the studies of mindfulness meditation [51]. Therefore, the temples are reasonable starting points of neuronal activity during both meditation and exercise. The correlation coefficients of the FFT-analyzed NIRS data differed significantly between the DLPFC and OFC in the meditation and exercise pair. This result showed that the NIRS channels most responsive to both meditation and exercise were localized more in the OFC than the DLPFC, indicating that the insular cortex (*i*.*e*., OFC) is an important region involved in both meditation and exercise.

An important difference between NIRS and other brain imaging systems, *e*.*g*., functional magnetic resonance imaging (fMRI), is that participants can maintain a sitting or standing position for NIRS, whereas they must assume a supine position for fMRI. Assuming supine position is thought to induce large changes in the autonomic nervous system [52]. With regard to the NIRS sampling time, neural activity triggers regional vasodilation in the brain. Although the mechanisms underlying activity-dependent vascular responses are complex and controversial, the extensive innervation of brain blood vessels suggests direct neuronal control of regional vasodilation [53]. This vasodilation mainly depends on the production of nitric oxide [54]. Previous studies demonstrated that the nitric oxide-induced vasodilation is observed with a time scale ranging from a few hundred milliseconds to a few seconds after stimulation [55]. Because the NIRS sampling time was 10 Hz, we can conclude that NIRS assesses brain function through the intact skull by detecting changes in blood hemoglobin concentrations associated with neural activity via vasodilation.

Mental refreshment or happiness in humans involves 5-hydroxytryptamine (5-HT) and oxytocin, among other neurotransmitters [56–58]. Both 5-HT and oxytocin are found in the insular cortex [59, 60]. Some studies report that 5-HT increases in participants who experience exercise-induced euphoria [61, 62]. In addition, intranasal administration of oxytocin boosts participants’ experience of specific positive emotions during meditation [63], and saliva oxytocin levels increase during meditation [64]. Although these articles did not refer to the insular cortex, the findings provide sufficient evidence that the same neurotransmitters or hormones play an important role in the same brain regions activated by meditation and exercise.

Previous NIRS studies examined brain regions that were activated when participants paid attention to their breathing movements in the lower abdomen during Zen meditation [65]. The oxyhemoglobin level increased symmetrically in the anterior prefrontal cortex when the participant’s attention was focused, and the participants reported a reduction in feelings of a negative mood compared with before the meditation session. On the other hand, alpha band activity is increased and theta band activity is decreased during and after the focused attention in EEG. These EEG changes correlated with a significant increase in blood 5-HT levels. Therefore, activation of the anterior prefrontal cortex and 5-HT system is considered to play an important role in the improvement of a negative mood. Measurements of 5-HT in blood should be examined in a future study. Another EEG study showed asymmetric changes in anterior brain activation during mindfulness meditation [66]. The meditators showed a significant increase in activation only in the left-side anterior temporal region. Thus, a left-right asymmetry of activated brain regions is likely a property of mindfulness. In the present study, we monitored function only in the forehead area of brain, because this area is uncovered by hair and easily measured with NIRS. Other apparatuses can examine various regions even in hair-covered areas.

Previous studies using EEG measurements revealed a peak at a specific EEG frequency in the prefrontal cortex and the left temporo-parietal area in participants with extensive experience in meditation [67]. Further, the EEG frequency varied with the type of meditation [68]. In the present study, all the participants were new learners of meditation, resulting in the demonstration that both meditation and exercise activated the same brain region. In the future, we will perform experiments similar to the EEG studies, and deepen our understanding of the activation of the OFC and DLPFC.

We finally discuss the limitation of our present study. Many researches using NIRS has not used the analysis method of power spectral density or FFT. Thus, FFT is brand-new for the analysis of NIRS data. As the first step in NIRS data analysis, we selected the FFT method rather than the power spectral density. Our NIRS (Hitachi ETG-4000) can set only 0.001, 0.01, and 0.1 frequency (Hz) as the cut-off value. We thought that the high pass filter of 0.001 Hz was the best, because the others may cutoff the essential frequency range of data. Hitachi ETG-4000 automatically obtains the 5-s moving average data to remove artifacts. Furthermore, as described earlier, we used the rectangular window for FFT. The divergence of the data is also known after FFT due to Gibbs phenomenon, which is caused by an inappropriate application of the window function to discontinuous graphs [69]. However, it is unlikely to be caused by Gibbs phenomenon, because we also take a 5-s moving average and the measured data are smooth. We believe that there are no problems associated with this issue in the post-FFT data.

In conclusion, the present study showed that the edges of the OFC are activated by both meditation and exercise. These regions are temporal areas reflecting insular cortex activity. Activation of the insular cortex by both meditation and exercise has been reported previously. The findings of our NIRS study implicate the involvement of this cortical region in the mental refreshment experienced by human beings when attention is focused on a specific body feature, such as breathing or muscle activity.

## Supporting information

S1 File (XLSX)
